# Tongue Thickness and Skin-to-Hyoid Distance as Ultrasonographic Markers of Difficult Airway in Patients Without Anticipated Difficulty: A Prospective Diagnostic Accuracy Study of 713 Adults †

**DOI:** 10.3390/healthcare14152277

**Published:** 2026-07-26

**Authors:** Sinan Mutlu, Hakan Küçükkepeci, Ece Özcan, Kadriye Serap Karacalar

**Affiliations:** 1 Department of Anesthesiology and Reanimation, Cizre Dr. Selahattin Cizrelioğlu State Hospital, 73200 Şırnak, Türkiye; ssinanmutlu@gmail.com; 2 Department of Anesthesiology and Reanimation, University of Health Sciences, Prof. Dr. Cemil Taşcıoğlu City Hospital, 34384 Istanbul, Türkiye; mdeceozcan@gmail.com (E.Ö.); skaracalar@yahoo.com (K.S.K.)

**Keywords:** difficult airway, airway ultrasonography, tongue thickness, skin-to-hyoid distance, difficult mask ventilation, difficult laryngoscopy, point-of-care ultrasound

## Abstract

**Highlights:**

**What are the main findings?**

- Increased tongue thickness and skin-to-hyoid-bone distance were independently associated with a difficult airway, whereas cricothyroid membrane length was not.
- Their negative predictive values were high but prevalence-dependent, with only modest likelihood ratios; adding them to clinical variables modestly improved discrimination.

**What are the implications of the main findings?**

- Anterior-neck ultrasonography may modestly lower the estimated probability of a difficult airway, rather than confirm one—complementing, not replacing, routine clinical screening.
- Cricothyroid membrane length showed no clinically meaningful discrimination in this cohort.

**Abstract:**

**Background/Objectives:** In patients without clinically anticipated difficulty, an unexpected difficult airway carries the greatest potential for harm, yet routine bedside tests exclude difficulty poorly, and evidence for point-of-care ultrasonography remains heterogeneous, with difficult mask ventilation rarely studied as a distinct outcome. We evaluated three rapid anterior-neck ultrasonographic parameters—tongue thickness (TT), skin-to-hyoid-bone distance (SHBD), and cricothyroid membrane length (CTML)—for their diagnostic performance and independent association with a difficult airway in adults without anticipated difficulty. **Methods:** In this prospective, single-center, diagnostic-accuracy study reported per STARD 2015 and STROBE, 713 adults (ASA I–III) underwent neutral-position measurement of TT, SHBD, and CTML by a sonographer blinded to the clinical assessment and airway-management outcomes. A composite difficult airway (difficult mask ventilation, laryngoscopy, or intubation) was assessed independently. Discrimination was evaluated by ROC analysis with DeLong comparisons, multivariable logistic regression, and bootstrap internal validation; *p*-values used Benjamini–Hochberg correction. **Results:** A difficult airway occurred in 197/713 patients (27.6%). TT and SHBD were associated with the composite outcome and all components (AUC 0.68–0.73) and remained independent after adjustment for age, sex, BMI, and Mallampati class; CTML discriminated poorly (AUC ≈ 0.55), with only marginal significance after correction. Negative predictive values were high (78–99%) but prevalence-dependent, with modest likelihood ratios. Adding TT and SHBD to clinical variables improved discrimination (AUC 0.69 → 0.78; DeLong *p* < 0.001). **Conclusions:** TT and SHBD show a limited but independent diagnostic signal that may complement, not replace, clinical screening; CTML showed no clinically meaningful discrimination in this cohort. Proposed cut-offs are exploratory and require standardized measurement, reproducibility testing, and external validation before clinical adoption.

## 1. Introduction

Securing and maintaining a patent airway is a fundamental responsibility of the anesthesiologist, and difficult or failed airway management remains an important and potentially preventable source of perioperative morbidity and mortality, including airway trauma, hypoxic brain injury, and death [1,2,3]. Reliable preoperative identification of patients in whom airway management is likely to be difficult is therefore central to safe practice, as it allows the appropriate equipment, skilled personnel, and rescue strategies to be prepared in advance [4,5]. This need is greatest in patients judged preoperatively to be low risk because the unanticipated difficult airway—where no preparation has been made—carries the highest potential for catastrophic harm; large registry data indicate that most difficult airways are not predicted by routine assessment [3,5].

Conventional bedside screening tests—chiefly the modified Mallampati classification, the thyromental and sternomental distances, and the upper-lip-bite test—are widely used but have limited and inconsistent diagnostic accuracy and in particular a poor negative predictive value for excluding a difficult airway [6,7,8]. Reported incidences of difficult laryngoscopy and intubation vary widely across populations and definitions, reflecting differences in case mix, laryngoscopic grading, and operator experience [7,8]. The modest and variable performance of any single test has prompted interest in both combined predictive models and more objective, anatomy-based measures.

Point-of-care ultrasonography (POCUS) has emerged as a noninvasive, rapid, repeatable, and radiation-free modality for evaluating the upper airway. Current expert guidance, including the 2022 American Society of Anesthesiologists practice guidelines and the 2025 Difficult Airway Society guidelines, now recognizes airway POCUS as a useful—though still optional—adjunct for airway assessment, cricothyroid membrane localization, and confirmation of tube placement [9,10]. A 2025 systematic review and meta-analysis of 60 studies comprising 10,580 participants confirmed both the growing clinical interest in airway POCUS and the substantial heterogeneity that currently limits it: the parameters studied, their measurement techniques, and their reported threshold values differ markedly between studies, difficult mask ventilation has been examined as a discrete outcome in comparatively few reports, and several proposed parameters remain inconsistently validated [11]. To be adopted into routine preoperative workflow, an ultrasonographic measurement should be simple, reproducible, quickly obtained, and feasible even for relatively inexperienced operators and in patients who cannot be optimally positioned.

Three anterior-neck parameters satisfy several of these practical criteria: tongue thickness (TT), skin-to-hyoid-bone distance (SHBD), and cricothyroid membrane length (CTML) can all be acquired rapidly with the head in the neutral position—an advantage for uncooperative patients and for those requiring cervical-spine protection. TT and SHBD have shown promise in earlier, predominantly smaller studies, but threshold values are not transferable because they depend critically on the precise tissue boundaries included in the measurement. CTML, by contrast, has scarcely been evaluated as a predictor of airway difficulty, having been studied mainly for front-of-neck access planning rather than for risk stratification. These measurements are intended to complement, not replace, routine clinical screening.

The aim of this prospective observational study was to evaluate the effectiveness of three preoperative anterior-neck ultrasonographic parameters—TT, SHBD, and CTML—as predictors of a difficult airway and its individual components (difficult mask ventilation [DMV], difficult laryngoscopy [DL], and difficult intubation [DI]) in adult patients without an anticipated difficult airway. To limit measurement ambiguity, TT was defined a priori to exclude the skin, subcutaneous fat, and geniohyoid muscle, yielding thresholds that are not directly comparable with reports using whole-tissue tongue measurements. We hypothesized that greater TT and SHBD would be associated with a difficult airway, and we additionally assessed whether CTML carried independent predictive value.

## 2. Materials and Methods

### 2.1. Study Design and Setting

This prospective, single-center, observational diagnostic-accuracy study was conducted in the Department of Anesthesiology and Reanimation, University of Health Sciences, Prof. Dr. Cemil Taşcıoğlu City Hospital, Istanbul, Türkiye, between 8 March and 21 April 2023. The study is reported in accordance with the STROBE statement for observational studies and, as a diagnostic accuracy study, the STARD 2015 guideline [12,13]. Adults undergoing elective or emergency surgery under general anesthesia with endotracheal intubation were screened, and all those fulfilling the eligibility criteria during the study period were enrolled, constituting a consecutive series. The study was not prospectively registered in a clinical-trial registry.

### 2.2. Ethics

The study was conducted in accordance with the Declaration of Helsinki and approved by the Clinical Research Ethics Committee of the institution on 8 March 2023 (protocol code E-48670771-514.99-210762828). Written informed consent for the anesthetic procedure and, separately, for the ultrasonographic measurements was obtained from all participants.

### 2.3. Participants

Eligible patients were adults (>18 years) of ASA physical status I–III, scheduled for general anesthesia with endotracheal intubation, who were cooperative and in whom a difficult airway was not clinically anticipated. Exclusion criteria were pregnancy; previous head or neck trauma or surgery; a documented history of, or a clinically anticipated, difficult airway; age < 18 years; inability to cooperate; obesity (BMI ≥ 35 kg/m^2^); and any anatomical deformity, trauma, tumor, or subglottic stenosis of the upper airway. Because the study population was defined as patients without anticipated difficulty, a modified Mallampati (MMP) class IV or an upper-lip-bite-test class 3 was treated a priori as a marker of an anticipated difficult airway and led to exclusion.

### 2.4. Preoperative Clinical Airway Assessment and Reference-Standard Conditions

Demographic data (age, sex, height, weight, BMI) and routine bedside airway measures were recorded preoperatively: mouth opening, thyromental distance, and sternomental distance (measured with a ruler); dental status (intact/missing or broken/dentures); MMP class (I–IV) [14]; and the upper-lip-bite test. Clinical airway assessment and intraoperative airway management—which together constituted the reference standard—were carried out by attending anesthesiologists spanning a wide range of clinical experience (1–30 years), deliberately reflecting routine practice so that the index test could be evaluated under real-world conditions. All clinicians performing the reference standard were blinded to the ultrasonographic measurements.

### 2.5. Ultrasonographic Measurements (Index Test)

**Figure 1:**
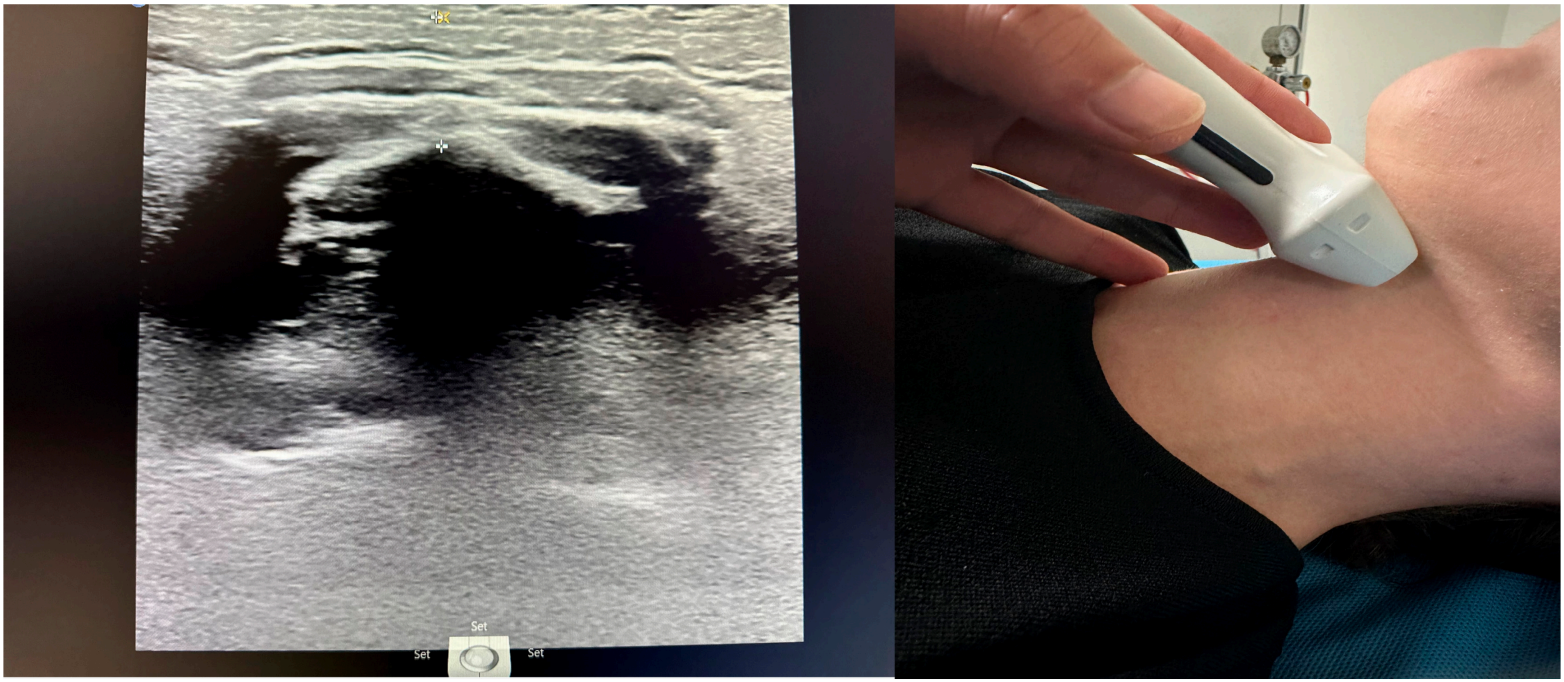
Ultrasonographic measurement of the skin-to-hyoid-bone distance (SHBD): linear probe placed transversely (**right**) and the corresponding sonographic view (**left**).

**Figure 2:**
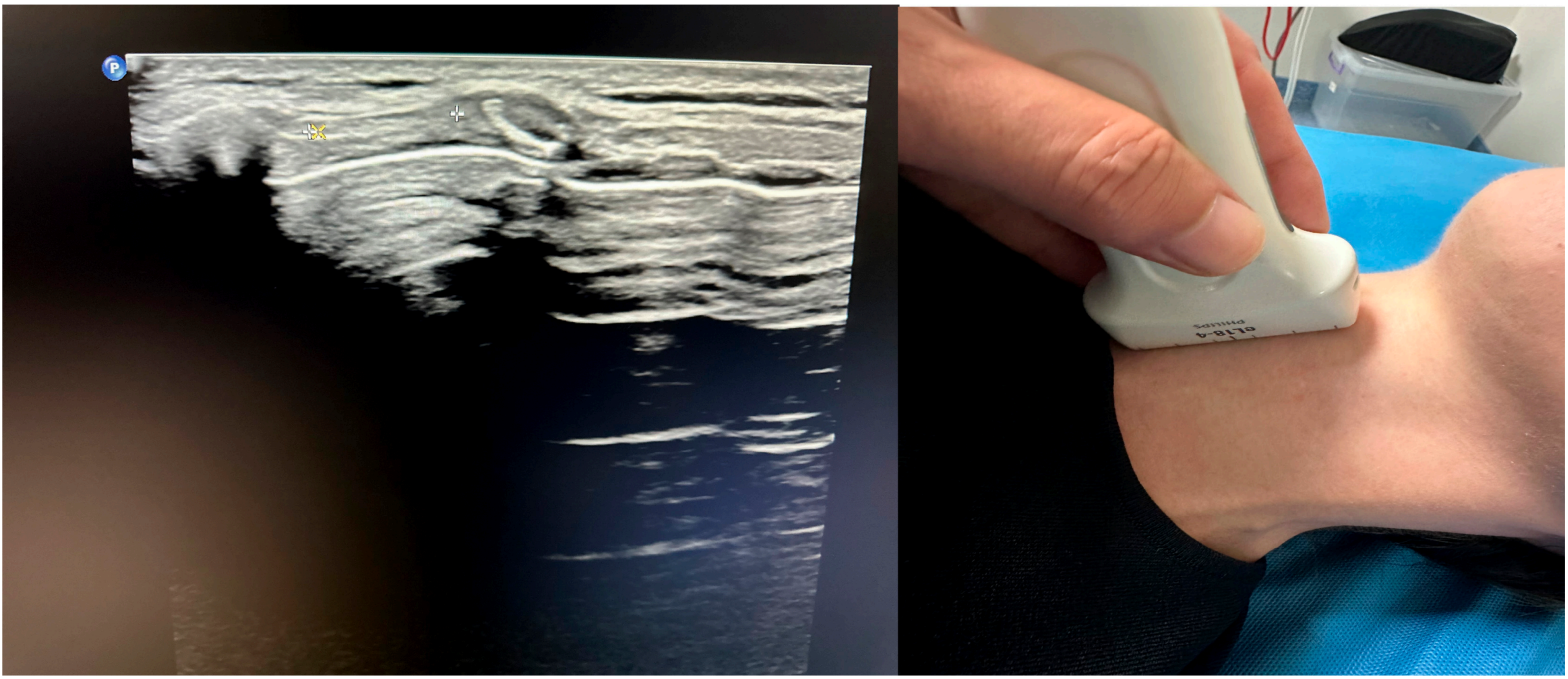
Ultrasonographic measurement of the cricothyroid membrane length (CTML): linear probe placed sagittally (**right**) and the corresponding sonographic view (**left**).

**Figure 3:**
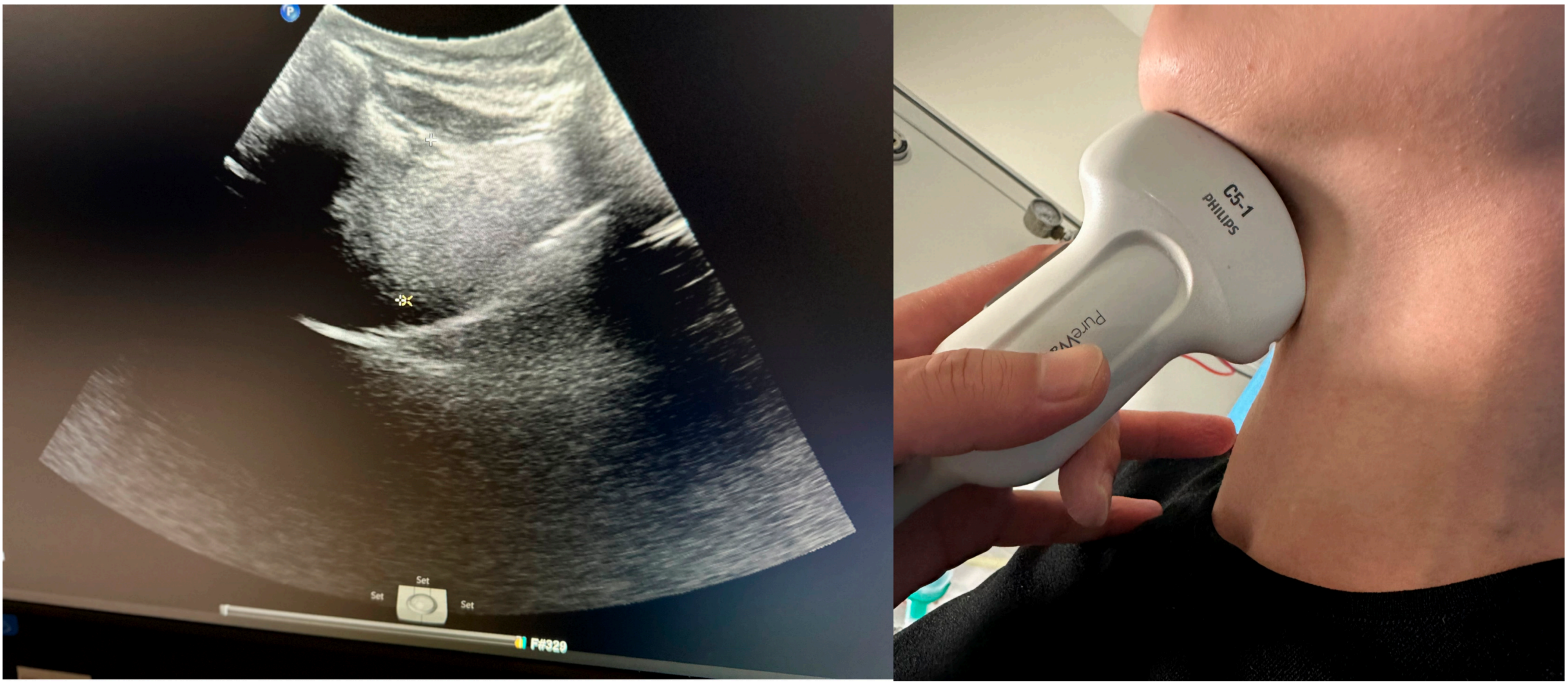
Ultrasonographic measurement of tongue thickness (TT): convex probe placed submentally (**right**) and the corresponding sonographic view (**left**).

To maximize reproducibility of the index test, all ultrasonographic measurements were obtained by a single experienced anesthesiologist in the preoperative holding area—with the patient supine and the head in the neutral position—before transfer to the operating room. The sonographer was blinded to the clinical airway assessment and to all subsequent airway-management outcomes, thereby avoiding incorporation and verification bias. A Philips Affiniti 70G system (Philips, Eindhoven, The Netherlands) was used with a 10–13 MHz linear probe and a 3–7 MHz low-frequency convex probe; all distances were recorded in centimeters:

- Skin-to-hyoid-bone distance (SHBD). With the linear probe in the transverse plane at the jugular notch, the hypoechoic inverted-U tracheal cartilages were identified. Without lifting the probe, it was slid cranially to the cricoid cartilage, then to the thyroid cartilage and thyrocricoid region, and finally—after slight probe tilting—to the hyperechoic, arched-bridge-shaped hyoid bone, where the SHBD was measured (Figure 1).
- Cricothyroid membrane length (CTML). The linear probe was placed in the sagittal plane at the suprasternal notch, and the hypoechoic tracheal rings (“string of pearls”) were followed cranially to the cricoid cartilage, thyroid cartilage, and cricothyroid membrane; the CTML was then measured along this sagittal view (Figure 2).
- Tongue thickness (TT). The 3–7 MHz convex probe was placed submentally in the midsagittal plane. With the patient supine and the head neutral, participants kept the mouth closed and the tongue relaxed, with the tip resting lightly against the incisors and without phonation. The image was optimized to display the entire tongue outline and then frozen. TT was defined as the maximum vertical distance from the tongue surface to the center of the oral-facing border of the geniohyoid muscle; the skin, subcutaneous fat, and geniohyoid muscle were deliberately excluded (Figure 3). This boundary definition was chosen because a parenchyma-only measurement expressed in centimeters is less susceptible to error from probe-applied pressure; consequently, the resulting thresholds are lower than and not directly comparable with those derived from whole-tissue tongue measurements.

No adverse events were associated with the ultrasonographic measurements.

### 2.6. Anesthesia and Outcome Definitions

Induction of general anesthesia followed routine institutional practice at the discretion of the managing anesthesiologist and was not standardized across participants. This pragmatic approach was adopted deliberately, so that the diagnostic performance of the index test could be evaluated under real-world conditions and across the full range of clinical experience present in routine practice (attending anesthesiologists with 1–30 years of experience; Section 2.4). Standard intraoperative monitoring was applied to every patient. In all patients, neuromuscular blockade was achieved with rocuronium at a weight-appropriate dose. Mask ventilation was assessed during induction, before neuromuscular blockade had fully developed, whereas laryngoscopy and intubation were undertaken only after an interval judged adequate for full onset. Quantitative neuromuscular monitoring was not used. Airway management was performed in the standardized sniffing position. The induction agents and the initial airway device and any adjuncts were selected by the managing anesthesiologist rather than fixed by protocol. The index test was obtained preoperatively in the holding area, and the reference standard was applied during induction of anesthesia on the same day, with no clinical intervention between them. All airway management and outcome grading were performed by an attending anesthesiologist other than the sonographer, who was blinded to the ultrasonographic data. Three components of airway difficulty were assessed, each defined a priori so that the technique-dependent maneuvers used to overcome difficulty were captured within the outcome grade itself rather than left as uncontrolled sources of variation:

- Difficult mask ventilation (DMV) was graded during induction with the Han scale [15], recorded according to a predefined protocol by the attending anesthesiologists. Because this scale incorporates the airway-management response into the grade—oral or nasopharyngeal airway insertion and adjuvants define the intermediate grades, and inadequate, unstable, or two-provider (two-handed) ventilation defines the higher grades—mask technique, adjunct use, and two-handed ventilation were reflected in the assigned grade rather than acting as separate confounders. Grades 1–2 were classified as easy and grades 3–4 as difficult.
- Difficult laryngoscopy (DL) was graded with the Cormack–Lehane classification [16] at laryngoscopy, the recorded value being the best laryngeal view obtained. The grade was recorded before any external laryngeal manipulation; other optimization maneuvers were not standardized and remained at the discretion of the managing anesthesiologist. A Cormack–Lehane grade was obtained in every patient, including the eight managed with first-line videolaryngoscopy, in whom the laryngeal view was scored on the same four-point scale. Grades 1–2 were classified as easy and grades 3–4 as difficult.
- Difficult intubation (DI) was assessed from a pre-defined record of the intubation method, the number of attempts, and the use of a stylet or other adjuncts. Intubation was coded as conventional direct laryngoscopy, direct laryngoscopy with a stylet, first-line videolaryngoscopy, or videolaryngoscopy with a stylet. The primary definition, based on the attempt-based criterion of the ASA practice guidelines [9], classified an intubation as difficult when it required three or more attempts or when tracheal intubation failed. Because a stylet or other adjunct may be used pre-emptively rather than only in response to difficulty, an adjunct-based definition—completion requiring a stylet or other adjunct, a supraglottic rescue, or failure—was retained only as a post hoc sensitivity analysis (Section 3.7). Elective first-line videolaryngoscopy performed without such adjuncts was not classified as difficult under either definition.

The primary composite outcome, difficult airway, was defined a priori as the presence of at least one of DMV, DL, or DI. This composite was pre-specified as the primary outcome because, in a patient without anticipated difficulty, the clinically important event is the occurrence of any form of airway difficulty and because each individual component is relatively infrequent; the overlap among the components and the diagnostic performance of each component separately are reported to show what drives the composite (Section 3).

Because airway management was intentionally not standardized, the reference standard reflects the variability of routine care. Two features were used to limit the resulting outcome misclassification: all index-test measurements were obtained by a single blinded operator (Section 2.5), separating index-test variability from outcome ascertainment, and each outcome was graded with an explicit scale whose categories already encode the principal technique-related maneuvers, as described above. Residual variability arising from non-standardized induction and from grading by multiple clinicians without a formal inter-rater reliability assessment is acknowledged as a limitation (Section 4).

### 2.7. Statistical Analysis

Statistical analyses were performed with IBM SPSS Statistics, version 26.0 (IBM Corp., Armonk, NY, USA). The additional analyses introduced at revision—multivariable logistic regression, DeLong comparison of ROC curves, bootstrap internal validation, and multiplicity correction—were performed in Python 3.12 (statsmodels, scikit-learn, and SciPy). Continuous variables are presented as mean ± standard deviation (and as median [minimum–maximum] where appropriate) and categorical variables as frequency (percentage).

A formal a priori sample-size calculation for diagnostic accuracy was not performed; instead, all eligible patients presenting during the study period were enrolled, and the resulting sample of 713 patients yielded precise estimates (standard error of the AUC ≈ 0.02 for the composite outcome). Analyses were performed on complete cases; patients with incomplete data were excluded (see participant flow), no index-test measurement yielded an indeterminate result, and missing values were not imputed. A post hoc sensitivity analysis for difficult intubation was conducted using the alternative adjunct-based definition (completion requiring a stylet or other adjunct, a supraglottic rescue, or failure).

The diagnostic performance of each parameter for the composite difficult airway and each component was assessed by receiver-operating-characteristic (ROC) analysis. For every parameter–outcome pair, the area under the curve (AUC) with its 95% confidence interval (CI) was calculated, and AUCs were compared using the DeLong test. The optimal cut-off was identified by the Youden index [17], and the corresponding sensitivity, specificity, predictive values, and likelihood ratios were calculated with their 95% confidence intervals (Wilson intervals for proportions; the log method for likelihood ratios), with predictive values derived using the observed prevalence of each outcome. Because three parameters were evaluated against four outcomes, *p*-values were corrected for multiple comparisons using the Benjamini–Hochberg false-discovery-rate method. To assess whether the ultrasonographic parameters were independent of clinical variables, a multivariable logistic regression was fitted for the composite outcome with age, sex, body mass index, and Mallampati class as covariates; the incremental value of ultrasonography was evaluated by comparing a clinical-only model with a model additionally including tongue thickness and skin-to-hyoid-bone distance (DeLong test). Internal validation used the bootstrap with 1000 resamples drawn with replacement from the full cohort; in each resample the entire modeling procedure was repeated, including refitting the logistic model and re-deriving the Youden-optimal cut-offs, and the fitted model was evaluated both on the resample (apparent performance) and on the original cohort (test performance). Optimism was estimated as the mean difference between apparent and test performance, and optimism-corrected estimates were obtained by subtracting this optimism from the apparent value; the distribution of the bootstrap cut-offs across resamples was summarized by the median and interquartile range. Calibration of the combined model was assessed by the calibration slope, the calibration intercept (calibration-in-the-large), and the Brier score, with an optimism-corrected calibration slope obtained by the same bootstrap procedure, and its clinical usefulness was examined by decision-curve analysis (net benefit versus threshold probability, compared with the treat-all and treat-none strategies). The proposed cut-offs are reported as exploratory. The composite outcome was pre-specified as primary. A two-sided *p* < 0.05 was considered statistically significant.

## 3. Results

### 3.1. Participant Flow and Characteristics

**Figure 4:**
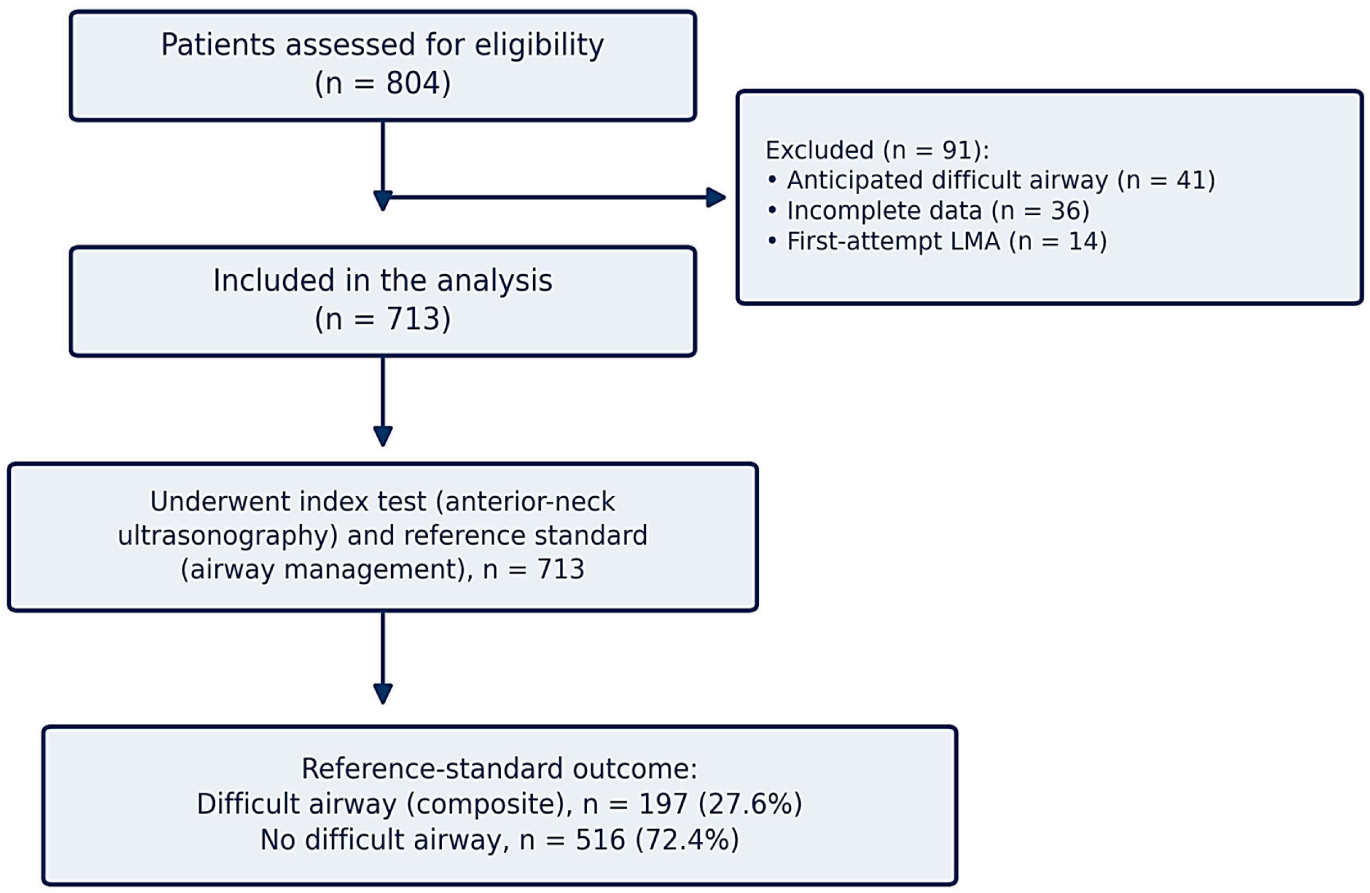
Flow of participants through the study (STARD diagram).

Of 804 patients screened, 91 were excluded—41 with an anticipated difficult airway, 36 with incomplete data, and 14 in whom a laryngeal mask airway was used at the first attempt—leaving 713 patients for analysis (Figure 4). These incomplete-data exclusions reflected one or more unrecorded entries on the clinical case-report form, rather than any failure of the ultrasonographic examination: interpretable images for all three parameters were obtained in every enrolled patient, and no participant was excluded because of ultrasound measurement failure or inadequate image quality. A field-by-field tabulation of the missing items was not compiled; because these 36 patients were excluded before analysis, their characteristics were not formally compared with those of the analyzed cohort, and a modest selection bias cannot be excluded (Section 4), although the excluded fraction was small (36/804, 4.5%) and the omissions were clerical rather than related to the index test or to airway anatomy. The mean age was 51.1 ± 16.0 years, and 403 patients (56.5%) were women. The ASA physical status was I, II, and III in 270, 433, and 10 patients, respectively. Mean height, weight, and BMI were 166.7 ± 8.3 cm, 75.1 ± 12.9 kg, and 26.9 ± 4.1 kg/m^2^. Surgery was elective in 703 patients and emergent in 10. Baseline characteristics are shown in Table 1.

### 3.2. Clinical Airway Assessment and Ultrasonographic Measurements

Consistent with the enrolment of patients without anticipated difficulty, no patient had a modified Mallampati class IV or an upper-lip-bite-test class 3, and the thyromental distance, sternomental distance, and mouth opening were within normal limits in all patients (Table 2). The mean ultrasonographic measurements were TT 3.68 ± 0.36 cm, SHBD 0.89 ± 0.15 cm, and CTML 0.79 ± 0.12 cm (Table 3).

### 3.3. Airway-Management Outcomes

Mask ventilation was easy in 333 patients (46.7%), normal in 282 (39.6%), and difficult in 98 (13.7%). Laryngoscopy was easy in 560 patients (78.5%) and difficult in 153 (21.5%). The trachea was intubated at the first attempt in 621 patients (87.1%), at the second in 65 (9.1%), at the third in 22 (3.1%), and at the fourth in 5 (0.7%); difficult intubation, defined as three or more attempts or a failed intubation, occurred in 27 patients (3.8%); no intubation failed (Table 4). Overall, a difficult airway occurred in 197 patients (27.6%).

### 3.4. Diagnostic Performance of the Ultrasonographic Parameters

**Figure 5:**
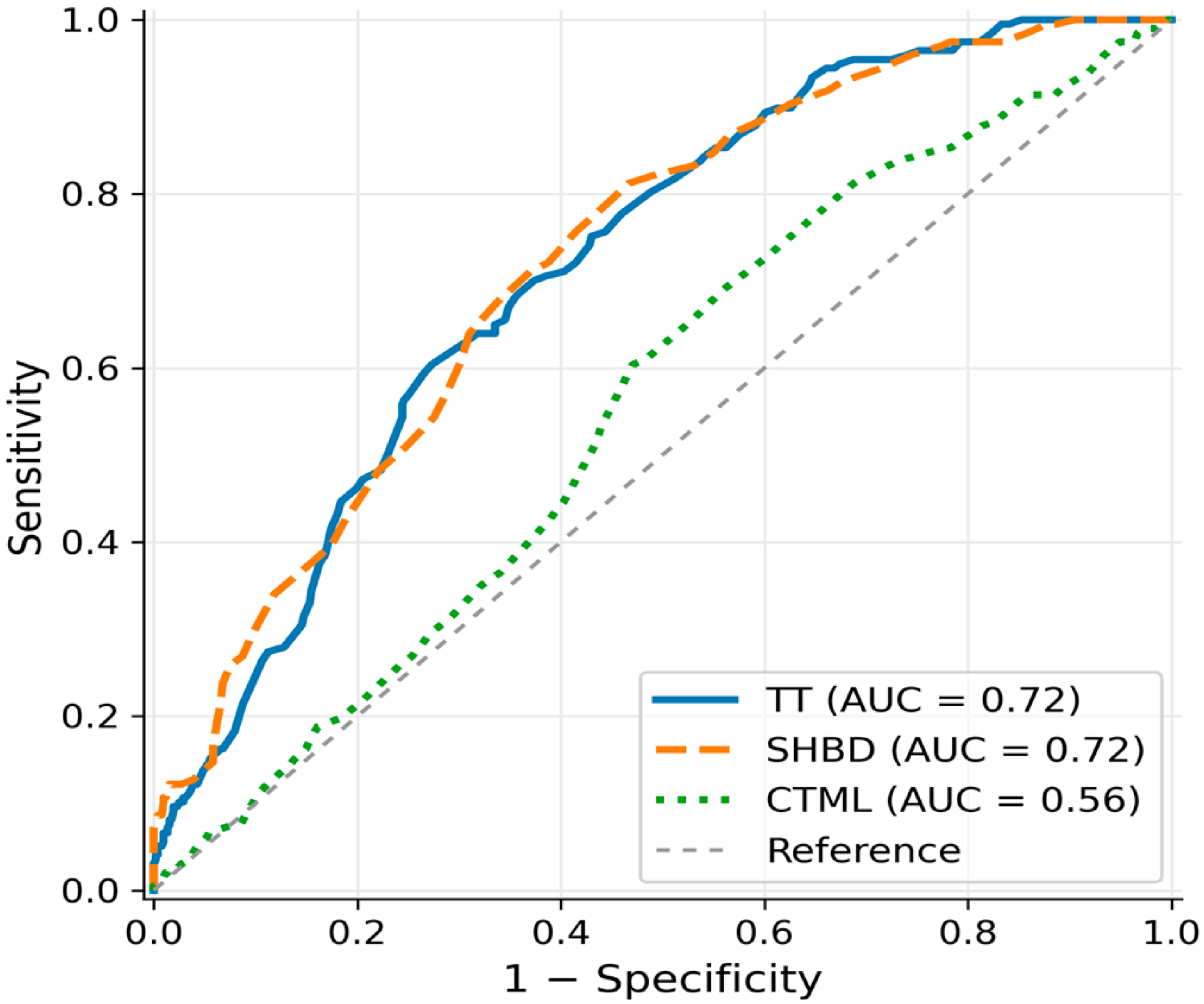
ROC curves of TT, SHBD, and CTML for the composite difficult airway.

**Figure 6:**
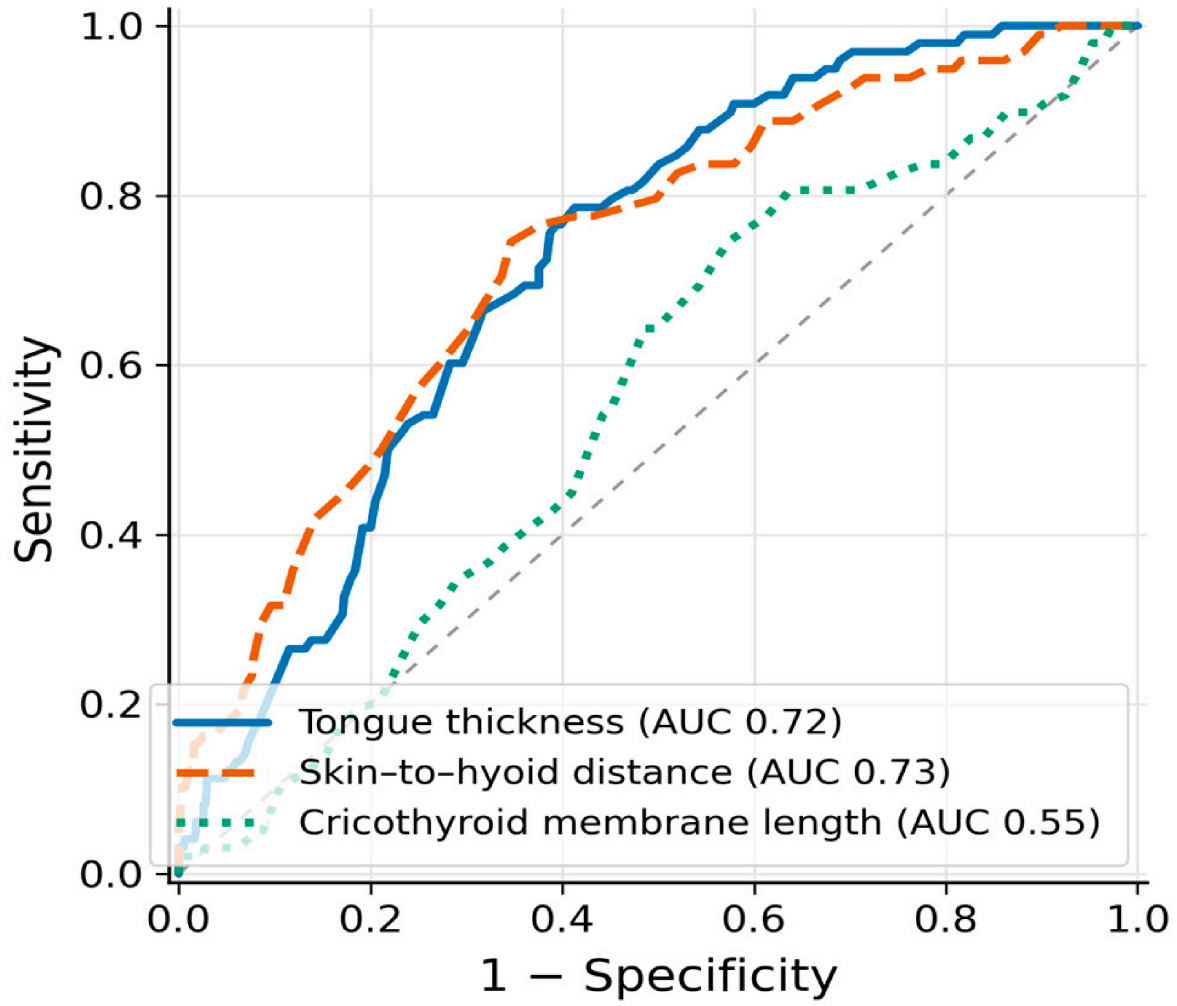
ROC curves of TT, SHBD, and CTML for difficult mask ventilation.

**Figure 7:**
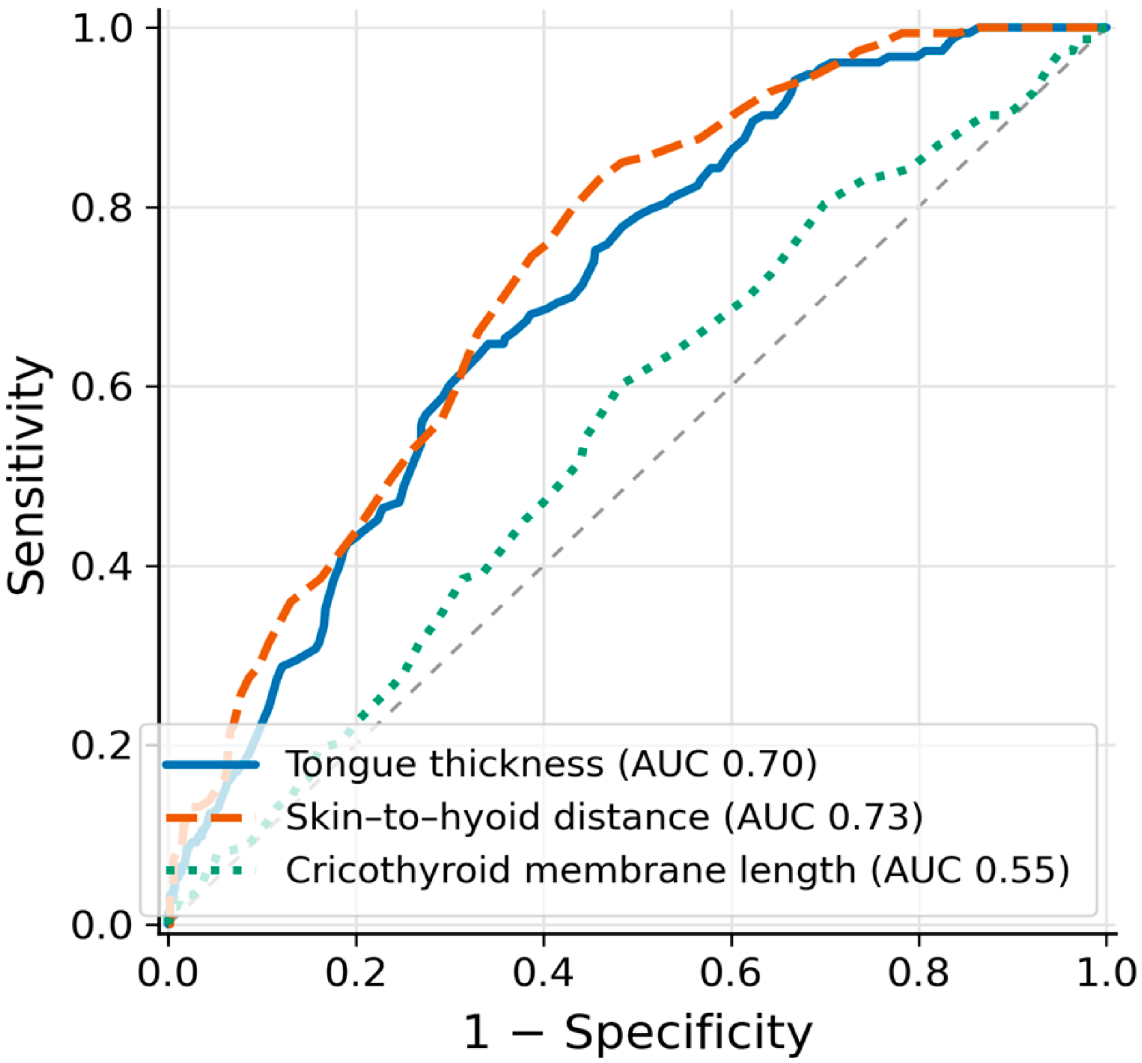
ROC curves of TT, SHBD, and CTML for difficult laryngoscopy.

**Figure 8:**
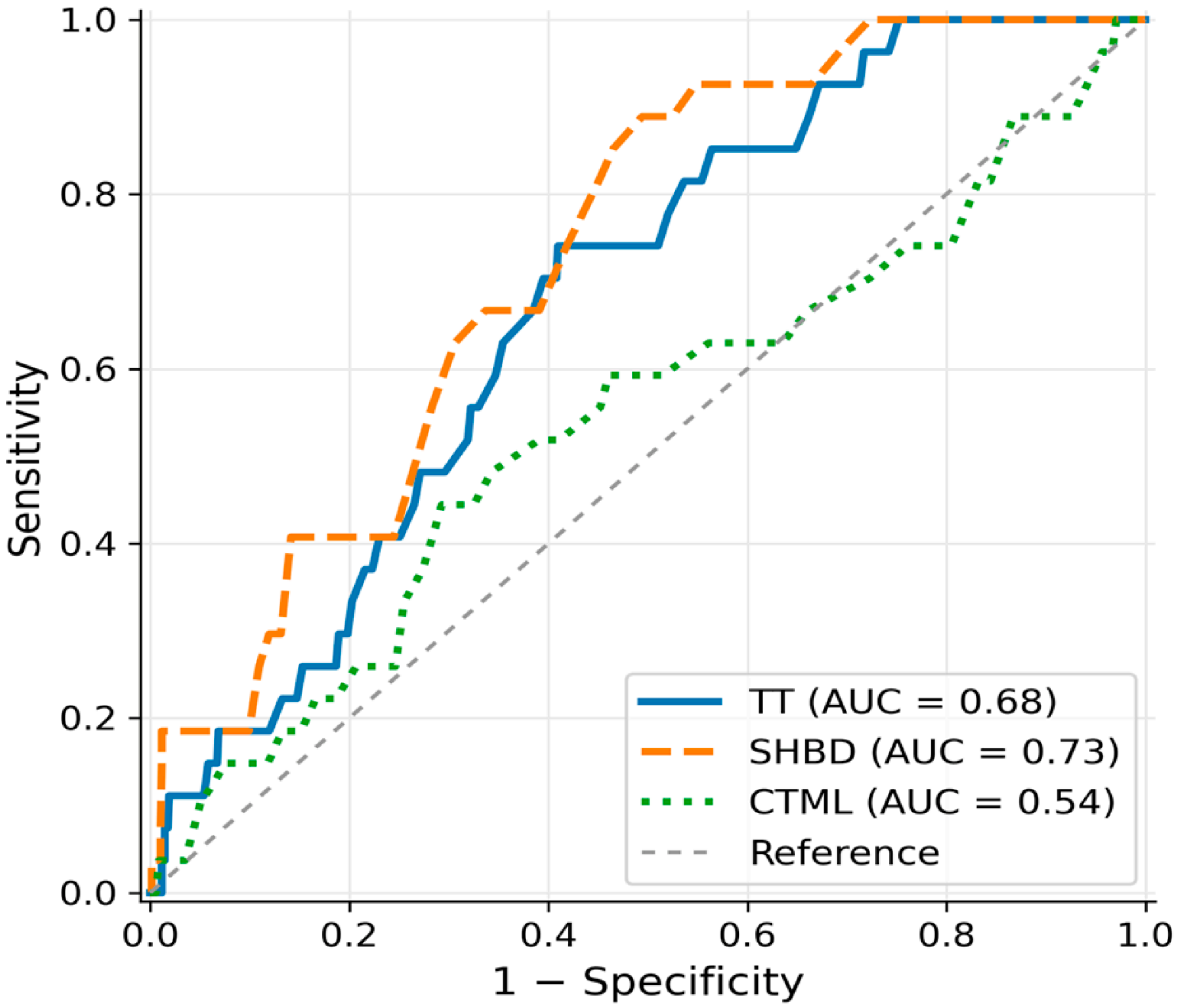
ROC curves of TT, SHBD, and CTML for difficult intubation.

TT and SHBD were significantly associated with the composite difficult airway and with all three components (AUC 0.68–0.73), whereas CTML showed poor discrimination (AUC ≈ 0.55). Negative predictive values were high (77.8–99.1%), whereas positive predictive values were low to moderate (5.6–45.8%) and were lowest for difficult intubation, reflecting its low prevalence. Likelihood ratios were modest (positive LR 1.20–2.21; negative LR 0.22–0.80), indicating only small shifts in post-test probability. Full diagnostic indices are presented in Table 5 (now including 95% confidence intervals for all indices and Benjamini–Hochberg-adjusted *p*-values), and the ROC curves are presented in Figure 5, Figure 6, Figure 7 and Figure 8. For the composite outcome, the areas under the curve for TT (0.72) and SHBD (0.72) did not differ from each other (DeLong *p* = 0.825) but were each significantly greater than that of CTML (0.56; both *p* < 0.001). After correction for multiple comparisons (Benjamini–Hochberg false-discovery-rate method), TT and SHBD remained significant for the composite outcome and for all three components, whereas the borderline associations of CTML did not survive correction for difficult mask ventilation (adjusted *p* = 0.073) or difficult intubation (adjusted *p* = 0.507) and were only marginally significant for the composite outcome (adjusted *p* = 0.018) and difficult laryngoscopy (adjusted *p* = 0.049).

### 3.5. Component Overlap Within the Composite Outcome

A difficult airway (composite outcome) occurred in 197 patients. Of these, 129 (65.5%) had a single isolated component—86 with isolated difficult laryngoscopy, 43 with isolated difficult mask ventilation, and none with isolated difficult intubation—whereas 55 patients had two concurrent components, and 13 had all three. Difficult laryngoscopy was therefore the most frequent single contributor to the composite, whereas difficult intubation never occurred in isolation, always co-occurring with difficult laryngoscopy or mask ventilation. Because the components differ in mechanism, frequency, and clinical importance and because difficult intubation never occurred in isolation, the composite is best regarded as a summary rather than as a single clinical entity; the individual components (Table 5) are therefore emphasized as the more clinically informative endpoints, and the composite, although pre-specified as the primary outcome, is interpreted cautiously as a summary measure of any airway difficulty.

### 3.6. Independent Association, Incremental Value, and Internal Validation

In a multivariable logistic regression for the composite outcome adjusted for age, sex, body mass index, and Mallampati class, both TT and SHBD remained independently associated with a difficult airway (TT: adjusted odds ratio [OR] 1.74 per standard deviation [SD], 95% CI 1.33–2.28, *p* < 0.001; SHBD: OR 2.04 per SD, 95% CI 1.59–2.62, *p* < 0.001), whereas CTML did not (OR 0.91 per SD, 95% CI 0.74–1.13, *p* = 0.39). Among the clinical covariates, only age was independently associated with the outcome (OR 1.02 per year, 95% CI 1.01–1.04, *p* < 0.001); sex, body mass index, and Mallampati class were not (Table 6).

Adding TT and SHBD to the clinical model increased the area under the curve from 0.688 (95% CI 0.646–0.730) to 0.781 (95% CI 0.745–0.816), a statistically significant improvement in discrimination (DeLong *p* < 0.001).

**Figure 9:**
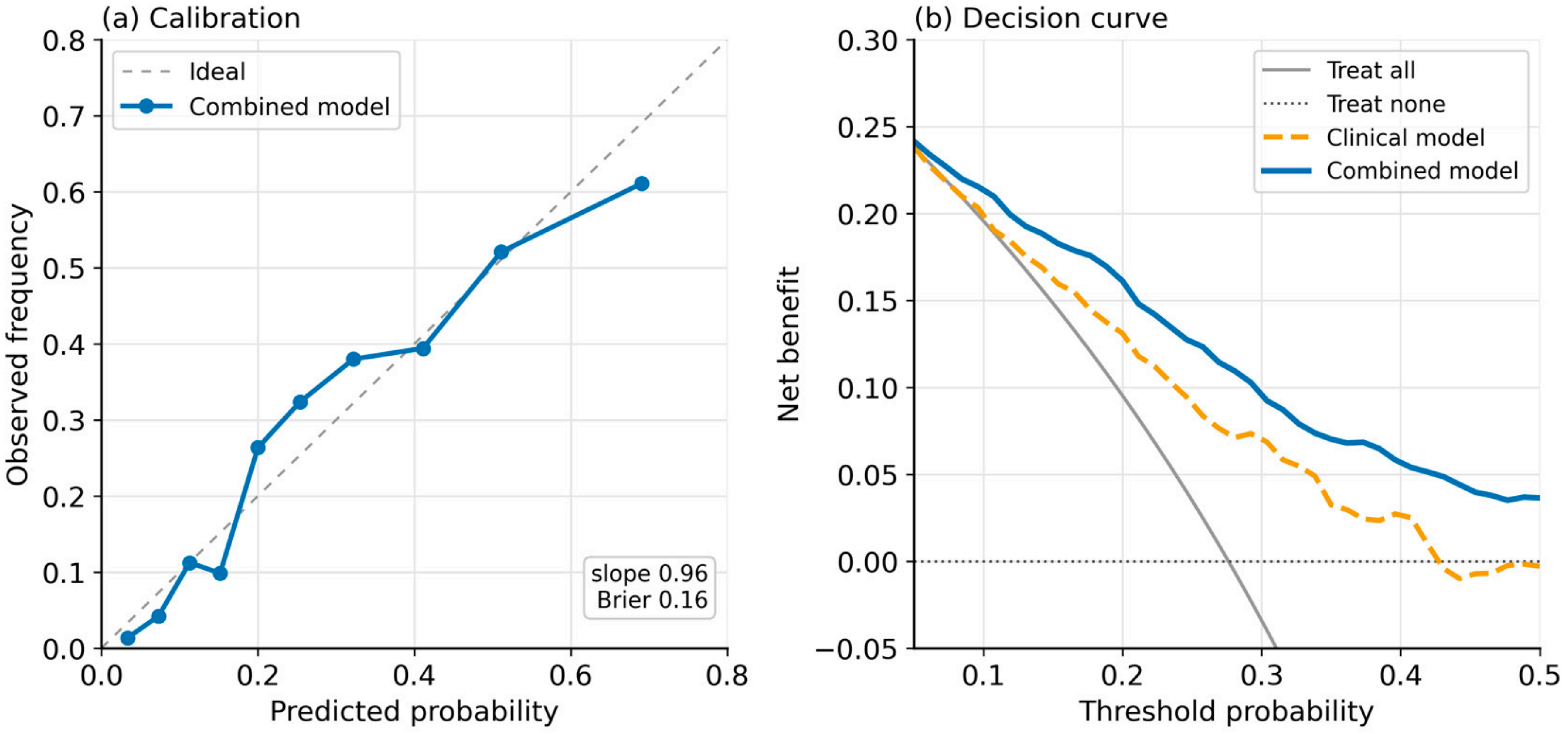
Calibration and clinical usefulness of the combined model (age, sex, body mass index, Mallampati class, tongue thickness, and skin-to-hyoid distance) for the composite difficult airway. (**a**) Calibration plot (deciles of predicted risk) with the bootstrap optimism-corrected calibration slope (0.96), calibration intercept (−0.02), and Brier score (0.16); the dashed line denotes perfect calibration. (**b**) Decision-curve analysis showing net benefit against threshold probability for the combined model, the clinical model, and the treat-all and treat-none strategies.

On bootstrap internal validation (1000 resamples, with the model refit and the cut-offs re-derived in each resample), the combined model showed minimal optimism: an apparent area under the curve of 0.781, an optimism of 0.008, and an optimism-corrected area under the curve of 0.773. The Youden-derived cut-offs were more variable across resamples (tongue thickness median 3.72 cm, interquartile range 3.66–3.80; skin-to-hyoid distance median 0.88 cm, interquartile range 0.86–0.91), so the proposed cut-offs are reported as exploratory. The combined model was well calibrated (optimism-corrected calibration slope 0.96 and calibration intercept −0.02; Brier score 0.16 versus 0.20 for a non-informative model), and in decision-curve analysis, it achieved a higher net benefit than the clinical model and the treat-all and treat-none strategies across the full range of clinically plausible threshold probabilities (for example, at a threshold probability of 0.25 the net benefit was 0.126 for the combined model versus 0.090 for the clinical model and 0.035 for treat-all), indicating that the gain in discrimination translated into a clinically meaningful net benefit (Figure 9). This incremental value is, however, relative to a parsimonious clinical model (age, sex, body mass index, and Mallampati class); because the cohort excluded patients with anticipated difficulty, other bedside screening tests were largely invariant and could not be included, so the gain may not extend to a comprehensive routine airway assessment.

### 3.7. Sensitivity Analysis for Difficult Intubation

Difficult intubation occurred in 27 patients under the primary, attempt-based definition (three or more attempts or failed intubation). The alternative adjunct-based definition (completion requiring a stylet or other adjunct, a supraglottic rescue, or failure) was met by 28 patients. These two definitions identify partly different patient groups: they overlapped in only 15 patients, with 12 patients meeting the attempt-based but not the adjunct-based definition and 13 the reverse. We therefore adopted the more conventional attempt-based definition as the primary outcome and retained the adjunct-based definition only as a sensitivity analysis. The diagnostic performance of the ultrasonographic parameters was similar under both definitions (all AUCs differed by no more than 0.02; for example, the SHBD AUC was 0.73 versus 0.72), although this similarity reflects the discrimination of the index tests and does not by itself establish the validity of either outcome definition.

## 4. Discussion

In this prospective study of 713 adults without an anticipated difficult airway, greater tongue thickness (TT) and a greater skin-to-hyoid-bone distance (SHBD) were associated with the composite difficult airway and with each of its components—difficult mask ventilation (DMV), difficult laryngoscopy (DL), and difficult intubation (DI)—with areas under the curve (AUCs) of 0.68–0.73. In contrast, cricothyroid membrane length (CTML) discriminated poorly (AUC ≈ 0.55) and reached significance only for the composite outcome and for DL. Across all outcomes the three parameters shared a characteristic profile: high negative predictive value (78–99%) but low positive predictive value and only modest likelihood ratios (positive LR 1.20–2.21; negative LR 0.22–0.80). The central message is therefore twofold: sub-mandibular and sub-hyoid sonographic measurements carry a real but limited predictive signal, and—because this cohort was restricted to patients without anticipated difficulty—their principal value lies in complementing, rather than replacing, conventional bedside assessment.

These findings align with recent syntheses of airway ultrasonography. A 2022 systematic review and meta-analysis found sub-hyoid soft-tissue distances to be the sonographic measures most able to flag difficulty even when routine clinical assessment is unremarkable, whereas anterior-neck thickness at the cricothyroid-membrane level predicted poorly [18]; a 2025 meta-analysis confirmed the growing utility of airway point-of-care ultrasonography but emphasized marked heterogeneity in the parameters, techniques, and thresholds reported [11]. Our results fit this frame closely: SHBD performed well in a deliberately low-risk cohort, CTML did not, and absolute cut-offs—including ours—are not transferable between studies.

The TT thresholds we identified (≈3.7–3.8 cm) are well below those of Yao and Wang (>6.1 cm) [19], Yadav et al. (>6.0 cm) [20], Xu et al. (>5.86 cm) [21], and Agarwal et al. (>5.8 cm) [22]. This difference is definitional rather than biological: we measured tongue parenchyma only—excluding skin, subcutaneous fat, and the geniohyoid muscle—whereas those studies measured whole-tissue thickness from the skin surface, and a 2025 study using yet another boundary reported intermediate values [23]. The direction of effect is nonetheless consistent—greater tongue bulk accompanies greater difficulty—and our finding that TT predicted DMV more strongly than DL echoes Lin et al. [24].

SHBD was our most consistent marker, achieving the highest AUCs for DMV and DI. This accords with prior reports linking increased hyoid-level soft-tissue distance to difficulty—Kaul et al. (>0.98 cm) [25], Wu et al. [26], Parameswari et al. [27], and Adhikari et al. [28]—and with Alessandri et al., who identified the skin-to-hyoid distance as the best sonographic predictor of DMV [29]. Negative reports (Fulkerson et al. [30]; Reddy et al. [31]) most likely reflect differences in the level and plane of measurement, body mass index, and laryngoscopy grading. The convergence of our larger sample with this positive evidence supports SHBD as a practical sub-hyoid marker of airway difficulty.

The weak performance of CTML is, given the cohort size, a genuine negative finding rather than a consequence of limited power. It aligns with the meta-analytic observation that cricothyroid-membrane-level thickness predicts difficulty poorly, whereas more cephalad sub-hyoid distances are informative [18]. The established clinical role of cricothyroid membrane sonography is anatomical localization for front-of-neck access and cricothyrotomy [32,33], not preoperative risk stratification; reporting this negative result is therefore useful, as it discourages adopting CTML as a predictive parameter.

Beyond univariable discrimination, our multivariable analysis showed that TT and SHBD were each independently associated with a difficult airway after adjustment for age, sex, body mass index, and Mallampati class, whereas CTML was not. Adding TT and SHBD to a clinical model improved discrimination (area under the curve 0.69 to 0.78; DeLong *p* < 0.001), indicating that anterior-neck ultrasonography captures information not contained in routine clinical variables and providing direct evidence that these parameters retain independent value. The incremental gain was nonetheless modest, and because both the clinical and the combined models were developed and evaluated in the same cohort, their apparent performance is likely optimistic and requires external validation before a combined ultrasonographic–clinical score could be recommended.

Difficult mask ventilation remains comparatively understudied with ultrasonography, and ours is among the larger datasets to address it; SHBD and TT showed the highest sensitivities for DMV in our cohort. Statistical significance, however, must be distinguished from clinical utility: although the AUCs for TT and SHBD were significant, their likelihood ratios were modest, producing only small shifts in post-test probability. Combined with high negative and low positive predictive values, this indicates that the parameters are better at lowering than at confirming the probability of difficulty—useful for reassurance and triage rather than as a stand-alone rule-in test and reinforcing a role that complements clinical screening. In the present study, combining tongue thickness and skin-to-hyoid distance with clinical variables already improved discrimination beyond a clinical model alone; prospective external validation of such combined models is therefore the logical next step. Practically, all three measurements were obtainable rapidly in the neutral head position, making them feasible when conventional tests cannot be performed—for example, in uncooperative patients or those requiring cervical-spine protection. The present findings do not by themselves justify the routine adoption of anterior-neck ultrasonography for preoperative airway-risk stratification, which would require the standardized measurement and external validation outlined below.

This study has several strengths. It was prospective, with a clear separation between the index test and the reference standard: the sonographer was blinded to both the clinical assessment and the airway-management outcomes, avoiding incorporation and verification bias, and all measurements were obtained by a single experienced operator, maximizing within-study measurement consistency. The reference standard was applied by attending anesthesiologists of widely varying experience, reflecting routine practice and supporting generalizability, and was ascertained completely, with no patient left unintubated. The sample is among the larger cohorts reported for this question, and the composite outcome was pre-specified.

Several limitations temper these findings. First, the optimal cut-offs were derived from the same dataset; although bootstrap internal validation indicated only small optimism, the reported sensitivities and specificities remain potentially optimistic, and external validation in an independent cohort is warranted before routine clinical implementation. Second, multiple comparisons were made (three ultrasonographic parameters across four outcomes); although *p*-values were corrected with the Benjamini–Hochberg false-discovery-rate method, the associations for CTML remained at most marginally significant after correction—for the composite outcome and difficult laryngoscopy only—and, given its poor discrimination, should be regarded as hypothesis-generating. Third, the reference standard was graded by many clinicians without a formal inter-observer reliability assessment. In addition, all ultrasonographic measurements were obtained by a single operator, so the inter- and intra-observer reproducibility of the index test—especially relevant for an operator-dependent modality and for the non-standard tongue-thickness boundary used here—could not be assessed; formal reliability testing (for example, the intraclass correlation coefficient) is a priority for future validation. Fourth, TT was measured with a non-standard boundary, limiting direct comparison with other reports. Fifth, because the cohort was restricted to patients without anticipated difficulty, several bedside screening tests were normal or had limited variation by design (the sternomental distance, mouth opening, and thyromental distance were essentially invariant, and the upper-lip-bite test had a restricted distribution); we therefore used a parsimonious clinical model comprising age, sex, body mass index, and Mallampati class, and a broader head-to-head comparison against the full range of clinical screening tests—recently undertaken in similar populations [23]—was not possible in this cohort. We also excluded obese patients, in whom airway ultrasonography has been evaluated separately [34]. Finally, the study was single-center, induction was not standardized, and the low prevalence of DI (3.8%) produced wide confidence intervals and a low positive predictive value for that outcome. Two further points bear on generalizability: the 36 patients excluded for incomplete case-report-form data were not analyzed and could not be compared with the study cohort, so a modest selection bias cannot be excluded (although these omissions were clerical and unrelated to the ultrasonographic examination); and, because only 10 of the 713 patients underwent emergency surgery, the diagnostic performance of the parameters could not be meaningfully compared between emergency and elective cases, leaving their behavior in emergency and trauma settings to be established.

Future work should focus on prospective external validation of thresholds using standardized measurement conventions, head-to-head and combined ultrasonographic-plus-clinical predictive models (for example, TT together with SHBD), formal inter-rater and intra-rater reliability studies (including intraclass correlation), evaluation in higher-risk populations such as obese and emergency or trauma patients, and the emerging role of artificial-intelligence-assisted image interpretation.

The observed incidence of a difficult airway (27.6%) and of its components (difficult mask ventilation 13.7%, difficult laryngoscopy 21.5%, and difficult intubation 3.8%) merits comment, given that the cohort was selected to be without anticipated difficulty. The difficult-intubation rate falls within the usually reported range of approximately 1.5–6%. The difficult-mask-ventilation rate lies at the upper end of the reported spectrum, which ranges from under 2% to about 15% depending on the definition used [35]; the Han grade-3 threshold applied here, which classifies any two-handed or two-provider ventilation as difficult, is comparatively inclusive and raises the rate relative to studies requiring inadequate or unstable ventilation. The difficult-laryngoscopy rate is high but not unprecedented, as reported Cormack–Lehane grade 3–4 incidences vary widely (from under 5% in large registry analyses to over 20% in some elective surgical cohorts) and are sensitive to operator experience and to the pragmatic, non-standardized induction used here. Because the composite necessarily exceeds any single component and combines outcomes that differ in mechanism, frequency, and clinical importance, it is interpreted cautiously as a summary measure, with the individual components emphasized as the more clinically informative endpoints.

## 5. Conclusions

In adults without a clinically anticipated difficult airway, increased preoperative tongue thickness and skin-to-hyoid-bone distance—measured by anterior-neck ultrasonography in the neutral head position—were associated with a difficult airway and with each of its components, whereas cricothyroid membrane length showed no clinically meaningful discrimination in this cohort. Because these measurements provide a limited but potentially useful diagnostic signal, with high negative predictive values but only modest likelihood ratios, their realistic role is to modestly lower the estimated probability of difficulty and to help triage patients rather than to serve as a stand-alone rule-in test; they are best used to complement, not replace, routine clinical screening. As the cut-off values are not yet standardized or externally validated, prospective multicenter validation using explicit measurement conventions, reproducibility (inter- and intra-observer) testing, and combined ultrasonographic-plus-clinical predictive models is needed before these parameters can be adopted for routine clinical use.

## Figures and Tables

**Table 1:** Demographic and clinical characteristics (n = 713).

Variable	Value
Age (years), mean ± SD	51.1 ± 16.0
Female/Male, n (%)	403 (56.5)/310 (43.5)
ASA I/II/III, n	270/433/10
Height (cm), mean ± SD	166.7 ± 8.3
Weight (kg), mean ± SD	75.1 ± 12.9
BMI (kg/m^2^), mean ± SD	26.9 ± 4.1
Hypertension, n (%)	227 (31.8)
Malignancy, n (%)	140 (19.6)
Diabetes mellitus, n (%)	138 (19.4)
Ischaemic heart disease, n (%)	80 (11.2)
Asthma/COPD, n (%)	36 (5.0)
Neurological disease, n (%)	21 (2.9)
Chronic kidney disease, n (%)	7 (1.0)
Surgery elective/emergent, n	703/10

**Table 2:** Routine clinical airway assessment (n = 713).

Test	Category	n (%)
Modified Mallampati	I/II/III/IV	231 (32.4)/426 (59.7)/56 (7.9)/0 (0)
Upper-lip-bite test	I/II/III	631 (88.5)/82 (11.5)/0 (0)
Dentition	Intact/Dentures/Missing-broken	471 (66.1)/91 (12.7)/151 (21.2)
Thyromental distance	>6.5 cm	713 (100)
Sternomental distance	>13.5 cm	713 (100)
Mouth opening	Full	713 (100)

**Table 3:** Ultrasonographic measurements (cm).

Parameter	Mean ± SD	Median	Min–Max
Tongue thickness (TT)	3.68 ± 0.36	3.68	2.66–6.05
Skin-to-hyoid-bone distance (SHBD)	0.89 ± 0.15	0.89	0.53–1.59
Cricothyroid membrane length (CTML)	0.79 ± 0.12	0.78	0.50–1.18

**Table 4:** Airway-management outcomes (n = 713).

Outcome	Category	n (%)
Mask ventilation	Easy/Normal/Difficult	333 (46.7)/282 (39.6)/98 (13.7)
Laryngoscopy	Easy/Difficult	560 (78.5)/153 (21.5)
Intubation attempts	1/2/3/4	621 (87.1)/65 (9.1)/22 (3.1)/5 (0.7)
Difficult intubation	Yes	27 (3.8)
Composite difficult airway	Yes	197 (27.6)
Failed intubation	Yes	0 (0)

**Table 5:** Diagnostic performance of the three ultrasonographic parameters for the composite difficult airway and its components.

Outcome (prev.)	Param.	Cut-Off (cm)	AUC (95% CI)	Sens. % (95% CI)	Spec. % (95% CI)	PPV % (95% CI)	NPV % (95% CI)	LR+ (95% CI)	LR− (95% CI)	*p*	p (adj.)
Difficult airway (27.6%)	TT	>3.80	0.72 (0.68–0.76)	60 (53–67)	73 (69–76)	45.8 (39.8–51.8)	82.8 (79.0–86.0)	2.21 (1.85–2.65)	0.54 (0.45–0.65)	<0.001	<0.001
	SHBD	>0.86	0.72 (0.69–0.76)	81 (75–86)	53 (49–58)	39.9 (35.2–44.8)	88.1 (84.1–91.3)	1.74 (1.55–1.95)	0.35 (0.26–0.48)	<0.001	<0.001
	CTML	>0.77	0.56 (0.51–0.60)	60 (53–67)	53 (49–57)	32.9 (28.2–37.9)	77.8 (73.1–81.8)	1.28 (1.11–1.48)	0.75 (0.62–0.91)	0.013	0.018
Difficult mask ventilation (13.7%)	TT	>3.71	0.72 (0.67–0.76)	78 (68–85)	58 (54–62)	22.8 (18.6–27.5)	94.3 (91.4–96.1)	1.86 (1.61–2.14)	0.38 (0.26–0.55)	<0.001	<0.001
	SHBD	>0.92	0.73 (0.67–0.78)	74 (65–82)	65 (61–69)	25.2 (20.7–30.7)	94.0 (91.5–96.0)	2.11 (1.80–2.47)	0.40 (0.28–0.56)	<0.001	<0.001
	CTML	>0.74	0.55 (0.50–0.61)	74 (65–82)	42 (38–46)	16.9 (13.7–20.8)	91.0 (87.3–93.9)	1.28 (1.12–1.46)	0.62 (0.44–0.88)	0.067	0.073
Difficult laryngoscopy (21.5%)	TT	>3.78	0.70 (0.65–0.74)	64 (56–71)	65 (61–69)	33.3 (28.2–38.9)	86.9 (83.3–89.8)	1.83 (1.55–2.16)	0.55 (0.44–0.69)	<0.001	<0.001
	SHBD	>0.87	0.73 (0.69–0.77)	83 (76–88)	54 (50–58)	33.0 (28.5–37.8)	92.1 (88.6–94.5)	1.80 (1.60–2.02)	0.31 (0.22–0.44)	<0.001	<0.001
	CTML	>0.77	0.55 (0.50–0.60)	60 (52–68)	50 (46–54)	24.7 (20.6–29.4)	82.1 (77.7–85.8)	1.20 (1.03–1.40)	0.80 (0.65–0.99)	0.041	0.049
Difficult intubation (3.8%)	TT	>3.76	0.68 (0.59–0.77)	74 (55–87)	59 (55–63)	6.6 (4.3–10.0)	98.3 (96.5–99.2)	1.81 (1.42–2.30)	0.44 (0.23–0.83)	<0.001	<0.001
	SHBD	>0.88	0.73 (0.65–0.81)	89 (72–96)	51 (47–54)	6.6 (4.5–9.6)	99.1 (97.5–99.7)	1.80 (1.54–2.10)	0.22 (0.08–0.64)	<0.001	<0.001
	CTML	>0.84	0.54 (0.42–0.66)	44 (28–63)	71 (67–74)	5.6 (3.3–9.6)	97.0 (95.1–98.2)	1.52 (0.98–2.35)	0.79 (0.56–1.10)	0.507	0.507

AUC, area under the ROC curve; CI, confidence interval; Sens., sensitivity; Spec., specificity; PPV/NPV, positive/negative predictive value; LR+/LR−, positive/negative likelihood ratio. PPV and NPV were derived from sensitivity, specificity, and the observed prevalence; LR is prevalence-independent. Cut-offs were defined by the Youden index. Confidence intervals are Wilson intervals for proportions and log-method intervals for likelihood ratios; the AUC CI is the DeLong interval. p (adj.) denotes the Benjamini–Hochberg false-discovery-rate-adjusted *p*-value across the twelve parameter−outcome comparisons.

**Table 6:** Multivariable logistic regression for the composite difficult airway (n = 713).

Variable	Adjusted OR (95% CI)	*p*
Age (per year)	1.02 (1.01–1.04)	<0.001
Male sex	0.73 (0.47–1.13)	0.16
BMI (per kg/m^2^)	1.03 (0.98–1.09)	0.22
Mallampati class (per class)	1.33 (0.96–1.85)	0.088
Tongue thickness (per SD)	1.74 (1.33–2.28)	<0.001
Skin-to-hyoid distance (per SD)	2.04 (1.59–2.62)	<0.001
Cricothyroid membrane length (per SD)	0.91 (0.74–1.13)	0.39

OR, odds ratio; CI, confidence interval; SD, standard deviation (tongue thickness 0.36 cm, skin-to-hyoid distance 0.15 cm, cricothyroid membrane length 0.12 cm). The reference category for sex is female. Ultrasonographic parameters are expressed per SD to aid interpretation.

## Data Availability

The data presented in this study are available from the corresponding author upon reasonable request. The data are not publicly available due to privacy and ethical restrictions.

## Abbreviations

The following abbreviations are used in this manuscript:

TT	Tongue thickness
SHBD	Skin-to-hyoid-bone distance
CTML	Cricothyroid membrane length
DMV	Difficult mask ventilation
DL	Difficult laryngoscopy
DI	Difficult intubation
AUC	Area under the ROC curve
ROC	Receiver-operating characteristic
PPV/NPV	Positive/negative predictive value
LR	Likelihood ratio
